# Knowledge, Anxiety, Depression, and Sleep Quality Among Medical Staff in Central South Areas of China During the Break of COVID-19: Does the Level of Hospitals Make a Difference?

**DOI:** 10.3389/fpsyt.2021.714870

**Published:** 2021-09-20

**Authors:** Haojun Yang, Ruiying Shi, Yunfang Chi, Zhihua Qiao, Yuanxia Wu, Ziqing Zhu, Bo Xiao, Li Feng, Hongxing Wang

**Affiliations:** ^1^Department of Neurology, Xiangya Hospital, Central South University, Changsha, China; ^2^Laizhou People's Hospital, Yantai, China; ^3^Department of Plastic and Aesthetic Surgery and Burns, Second Xiangya Hospital, Central South University, Changsha, China; ^4^Department of Rehabilitation, Xiangya Hospital, Central South University, Changsha, China

**Keywords:** knowledge, anxiety, depression, sleep quality, medical staff, COVID-19

## Abstract

**Purpose:** To evaluate the knowledge, anxiety, depression, and sleep quality toward COVID-19 among Chinese medical staff from tertiary and basic-level hospitals in central south areas of China.

**Method:** A structured questionnaire was composed of Demographic and clinical characteristics of medical staff, Knowledge toward COVID-19 including epidemiology and clinical manifestations, The Self-rating anxiety scale (SAS), Center for Epidemiologic Studies Depression Scale (CES-D), and The Pittsburgh Sleep Quality Index (PSQI). It was administered to medical staff from tertiary hospitals (Group A) (*n* = 407) and basic-level hospitals (Group B) (*n* = 388) during February 2020 and May 2020.

**Results:** Medical staff in group A had a stronger knowledge toward COVID-19 than group B (23.69 ± 5.83 & 18.15 ± 6.35, *p* < 0.001). Mild anxiety symptoms were found in both groups. The SAS scores (Mean ± SD) of group B were 58.87 ± 10.17, which was significantly higher than that of group A (52.59 ± 12.09, *p* < 0.001). There were no significant differences in CES-D scores between the two groups (*p* = 0.981). The mean score of total PSQI in group B (8.41 ± 3.03) was statistically higher than that of group A (7.31 ± 3.74, *p* < 0.001). Additionally, the scores of sub-components of group B, including subjective sleep quality, sleep latency, sleep disorder, sleeping medication use and daytime dysfunction, were significantly higher compared to Group A (*p* < 0.05).

**Conclusions:** Our study showed greater anxiety, more severe depression and poorer sleep quality among medical staff in central south areas of China during the COVID-19 outbreak. Additionally, compared to the tertiary hospital group, medical staff from basic-level hospitals had poorer knowledge toward COVID-19 and worse mental health conditions. In addition, residence, specialty, title and education level may also be factors of knowledge of COVID-19 and psychiatry problems. In light of this information, more attention should be paid to early identification and intervention of symptoms of anxiety and depression in susceptible medical staff from the basic-level hospitals.

## Introduction

The 2019 coronavirus disease (COVID-19) epidemic, which is the largest outbreak of atypical pneumonia since the severe acute respiratory syndrome (SARS) outbreak in 2003, is still a global health threat by far (1). The outbreak was first revealed in Wuhan City, Hubei Province, in late December 2019 when clusters of pneumonia cases of unknown etiology were found to be related to epidemiologically linked exposure to a seafood market and untraced exposures (2). Compared with SARS, COVID-19 has the characteristics of a long incubation period, no obvious upper respiratory symptoms, and strong infectivity (3). The COVID-19 outbreak has been declared by the World Health Organization (WHO) as a public health emergency of international concern on 30th January 2020, and a pandemic disease on 11th March 2020 (4). Globally, 13th August 2021, there have been 205,338,159 confirmed cases of COVID-19, including 4,333,094 deaths, reported to WHO (5).

Since the outbreak, the Chinese government has implemented strict public health measures against the spread of COVID-19 and dispatched medical staff from all over the country to support the first line of Hubei epidemic situation (6). A lockdown with travel restrictions was imposed on Wuhan on 23th January 2020, which was an unprecedented measure to restrict the spread of the virus. The quarantine was extended to other provinces and cities within days, affecting more than 50 million people in total. As of the end of data collection, there had been 84,565 confirmed cases in China, accompanied by a daily maximum of 15,152 diagnoses (5). It was a remarkable fact that the epidemic of the central south regions was the most serious in China, especially in Hubei province. At the same time, according to the published data from Wuhan, the bed occupancy rates in nearly all the tertiary hospitals were above 90%. In other words, medical staff were under both the heavy work pressure and psychological pressure of worrying about being infected (7). Previous researches indicated profound and wide range of psychosocial impacts on people at the individual, community, and international levels during the outbreak, which could not be ignored by us. Many individuals stayed at home and socially isolated themselves to prevent being infected, leading to a “desperate plea” (8, 9).

In addition, medical staff may also develop psychiatric disorders during the epidemic. During the SARS-CoV outbreak in Singapore, nearly 27% of health care workers reported psychiatric symptoms in 2003 (10). Moreover, post-traumatic stress disorder symptoms were found in medical staff that performed MERS-related tasks during the Korean outbreak in 2015. Studies during the Ebola outbreaks in Sierra Leone in 2014 and the Democratic Republic of the Congo in 2018 indicated those who were in direct contact with infected patients had higher levels of anxiety and the impact of stigma (11). Also, emergency professionals showed more severe post-traumatic stress disorder (PTSD) symptoms than staff in the psychiatric ward because of the feeling of interpersonal isolation and the fear that they would transmit the virus to their families (10). Medical staff also stated that the shortage of masks and health equipment made them more worried about being infected, and the use of heavy protective suits and N95 masks made communication between staff members difficult with related psychological distress (10). During the COVID-19 emergency, medical staff in China have dealt with a high risk of infection and inadequate protection from contamination, frustration, discrimination, patients with negative emotions, overwork, isolation, and a lack of contact with relatives (12). Recent studies revealed mental health problems, such as anxiety, depressive symptoms, insomnia and fear, among Chinese medical staff under such high work pressure. These mental health problems not only affected the attention, understanding and decision-making capacity of medical staff but also had a lasting effect on their overall well-being (12, 13). Psychological symptoms of COVID-19 on medical staff have been studied in previous researches. It was worth mentioning that several studies suggested that specific demographic characteristics may both affect the knowledge, attitudes as well as mental status of medical staff (14–16). Bhagavathula et al. indicated a correlation between certain demographic characteristic, such as age and occupation, and both inadequate knowledge and worse mental status toward COVID-19 (17). Besides, previous studies found hospital levels were related to their health workers' attitudes and knowledge toward certain diseases, such as epilepsy (18). However, there is still no research on whether the level of hospitals would affect the knowledge and mental status toward COVID-19 among medical staff in China. We assumed that the level of the hospitals could affect the knowledge and mental status toward COVID-19 among medical staff in China at the early stage of COVID-19. Therefore, the aim of the present study is to comprehensively evaluate the knowledge and mental status toward COVID-19 among medical staff in central south regions of China, and analyze whether they are related to demographic characteristics, especially the hospital levels.

## Materials and Methods

This cross-sectional questionnaire-based study was conducted between February 2020 and May 2020, which was a random sampling and performed after approval from the Ethics Committees of the Xiangya Hospital, Central South University. The purpose of the study was explained to the participants prior to distributing the questionnaire and the participants were required to answer the questionnaire without any intervention by the external factors, such as noises, hints and suggestions from others. Written consents were obtained and all questionnaires were administered anonymously. In order to reduce the risk of COVID-19 infection caused by face-to-face contact, all the participants in our study were enrolled via online questionnaire named Wenjuanxing, a platform providing functions equivalent to Amazon Mechanical Turk. The questionnaire link was distributed by the listed authors and some volunteers.

### Study Population

Medical staff were classified into two groups according to the level of hospitals they worked in based on the Hospital Classification Standards in China. General hospitals in China are categorized into three levels: the first-level hospitals should provide the basic medical care, prevention, rehabilitation, and health care services in small or medium-sized towns (18), the second-level hospital hospitals have to provide diagnosis and treatment of common and frequently occurring diseases, receiving referral patients from primary medical institutions and tertiary hospitals and undertaking teaching, training and scientific research tasks (18, 19), and the third-level hospitals are responsible for providing the maximum range of medical knowledge and technical infrastructure in diagnostics and treatment of almost all diseases (18, 20). In this study, the first-level and second-level hospitals were considered as basic-level hospitals and the third-level hospitals were considered as tertiary hospitals.

Group A comprised of medical staff from some tertiary hospitals and Group B comprised of medical staff from basic-level hospitals. The Level III hospitals selected by our study were regional medical centers, representing the large geographical and socio-economical parts of the Central South Areas of China, including Hunan, Hubei, Guangdong and Guangxi Provinces. Three to four tertiary hospitals were randomly chosen in each of these provinces. Finally, a total of 12 tertiary hospitals agreed to participate in this study. Three to four basic-level hospitals were randomly selected in each of all four regions (Center, North, Southwest, and Southeast) to get a representative view and mental status of medical personnel from multitudinous parts. Among the 12 invited hospitals, 8 different basic-level hospitals agreed to participate, which were usually located in rural or remote mountainous areas. Concerns of personal information disclosure was the main reason for the refusal. In addition to the willingness to participate in the research, the inclusion criteria for doctors and nurses with different specialties in hospitals was to be actively practicing at least a 6-month work experience. Participants were required to be over 18 years old and were not infected with SARS-CoV-2 during the pandemic break. Individuals who had a history of neurological disease, chronic physical disease, alcohol, or caffeine addiction were excluded from this study. In addition, participants during pregnancy or lactation had been excluded from this study.

### Assessment Tools

The questionnaire was composed of five blocks as follows: (1) Demographic and clinical characteristics of medical staff, (2) Knowledge toward COVID-19 including epidemiology and clinical manifestations, (3) The Self-rating anxiety scale (SAS), (4) Center for Epidemiologic Studies Depression Scale (CES-D), and (5) The Pittsburgh Sleep Quality Index (PSQI).

#### Data Collection Form

The Data Collection Form, a detailed interview form with questions about the general information of the participants, was prepared by the researchers for the purpose of this study. Age, gender, residence, occupation, specialty (Infectious, respiratory, emergency department or ICU, and others), title (Resident: Under training, no qualified independent practice; Attending Physician: Completed training, independent practice; Professor: Completed training, independent practice for more than 10 years with high level) and education level were included in the form.

#### Knowledge Toward COVID-19

The items in the second domain were extracted from the Guidelines for the Diagnosis and Treatment of 2019-nCoV Infection by the National Health Commission (Trial Version 5), which included epidemiological and clinical manifestations. Knowledge related to COVID-19 was assessed by 7 items, consisting of one choice question and six multiple choice questions where the respondent may only choose a single answer or choose multiple answers (21). Each question was worth 5 points. Points were only scored when the correct options were completely selected and no score would be awarded for a wrong or missed selection. This section was evaluated by scores and the percentage of the correct answers chosen by the participants (22).

#### The Self-Rating Anxiety Scale

The SAS was introduced by Zung in 1971 for measuring scate anxiety which was a transitory emotional state or condition of the human organism that is characterized by subjective, consciously perceived feelings of tension and apprehension and heightened autonomic nervous system activity (23). The Chinese version of SAS has been verified to have high internal consistency with Chinese population, whose Cronbach alphas was 0.931. There are 20 items in the scale, with 15 forward grading questions and 5 reverse (24). The total scores of the SAS was 1.25 multiplied by the sum of the scores of the 20 items. The cut-off score was 50, of which 50–59 were classified as mild anxiety, 60–69 were classified as moderate anxiety, and more than 69 were classified as severe anxiety.

#### Center for Epidemiologic Studies Depression Scale

The CES-D, developed by L.S. Radloff, was a tool for preliminary screening in ordinary people, to assess the frequency of depression symptoms (25). The Chinese version of CES-D has been tested and demonstrated good validity and reliability in general Chinese populations, whose Cronbach alphas was 0.90 (26). CES-D contains 20 items, four of which were reversely scored. The total score <15 indicates no depression symptoms, >16 indicates possible depression symptoms, and >20 indicates depression symptoms.

#### Pittsburgh Sleep Quality Index

The PSQI, consisting of 7 subcomponents in 18 questions, was developed by Buysse et al. (27). The Chinese version of the PSQI has been validated (Cronbach's alpha 0.87–0.94) (28). The 7 subscales are comprised of Subjective Sleep Quality, Sleep Latency, Sleep Duration, Sleep Efficiency, Sleep Disorder, Sleeping Medication Use, and Daytime Dysfunction. The total score ranges from 0 to 21, with higher scores indicating worse sleep quality. Poor sleep quality was defined as a total score of 7 or more in accordance with previous studies (29).

### Statistical Analysis

All demographic data were analyzed descriptively. Continuous data was presented as means and standard variations (Mean ± SD) and nominal data was presented as frequencies and percentages. The differences between the mean scores in demographic characteristics and each items was tested with two independent sample *t*-test. The Chi-Square test was used for comparison of groups regarding categorical variables. According to the variance analysis and a univariate linear regression model, the scores of SARS, CES-D, and PSQI among medical staff involved in this study were correlated with demographic characteristic. The remaining explanatory variables that were statistically significant were considered for the multivariate model for the mental status of medical staff. Statistical analysis was performed using the SPSS Version 24.0.0.0 (IBM, USA) and *p* < 0.05 was considered statistically significant.

## Results

### Demographic Data of Samples

A total of 433 medical staff in tertiary hospital were approached with 26 (6.0%) refusing to be interviewed and 407 (94.0%) agreeing. A total of 429 individuals in basic-level hospitals were approached. Out of these, 41 (9.6%) refused and 388 (90.4%) agreed. A lack of time and concerns of personal information disclosure were most frequently mentioned as a reason for the refusal in both groups. Table 1 showed a similar percentage of gender, age, residence, occupation, specialty, and title in two groups. Education level was the only significant difference between the two groups via the analysis of variance (*p* < 0.001).

**Table 1:** Demographic characterists of the study population.

**C**	**Tertiary hospital *n* (%)**	**Basic-level hospital (%)**	*P* ^*^
***N***	407 (51.19)	388 (48.81)	
**Gender**			
Male	165 (40.54)	167 (43.04)	0.475
Female	242 (59.46)	221 (56.96)	
**Age (years old)**			
18–25	39 (9.58)	46 (11.86)	0.300
26–35	141 (34.64)	135 (34.79)	
36–45	161 (39.56)	147 (37.89)	
≥46	66 (16.22)	60 (15.46)	
**Residence**			
Urban	265 (65.11)	236 (60.82)	0.211
Rural	142 (34.89)	152 (39.18)	
**Occupation**			
Doctor	217 (53.32)	201 (51.80)	0.669
Nurse	190 (46.68)	187 (48.20)	
**Specialty**			
Infectious, respiratory, emergency department or ICU	60 (14.74)	62 (15.98)	0.628
Others	347 (85.26)	326 (84.02)	
**Title**			
Resident	131 (32.19)	149 (38.40)	
Attending physician	236 (57.98)	201 (51.80)	
Professor	40 (9.82)	38 (9.79)	
**Education level**			
Technical secondary or below	1 (0.24)	58 (14.95)	** <0.001**
Junior college	61 (14.99)	190 (48.97)	
Undergraduate or above	345 (84.77)	140 (36.08)	

* *Statistical significance (p <0.05) is indicated in bold*.

### Knowledge Toward COVID-19 in Two Groups

Regardless of the total scores obtained when it was completely correct or the selection rates of the correct options, the knowledge toward COVID-19 among medical staff in tertiary hospitals was better than basic-level hospitals group (Table 2, *p* < 0.05). And the multiple linear regression suggested two predictors could explain 17.4% of the knowledge scores (*R*^2^ = 0.174, *F* = 24.878), including hospital level (β = −0.400, *p* < 0.001), and education level (β = 0.057, *p* = 0.05) (Table 3).

**Table 2:** Knowledge related to COVID-19 including epidemiology and clinical manifestations.

**Question**	**Frequency of YES answer (%)**		***P***
	**Group A *n* (%)**	**Group B *n* (%)**	
**Total scores**	**23.69** ***±*** **5.83**	**18.15** ***±*** **6.35**	** <0.001** ^***^
**Which one do you think is the name of the virus occurred first in Wuhan?**	**2.59** ***±*** **2.50**	**1.7** ***±*** **2.38**	** <0.001** ^***^
SARS-CoV-2^***^	211 (51.84)	134 (34.54)	** <0.001**
COVID-19^**^	184 (45.21)	223 (57.48)	**0.001**
MERSr-CoV^*^	12 (2.95)	24 (6.18)	**0.021**
Ebola virus^**^	0 (0)	7 (1.80)	**0.006**
**Which ways do you think are the distribution of SARS-CoV-2?^§^**	**2.79** ***±*** **2.48**	**2.50** ***±*** **2.50**	**0.022** ^*^
Droplet transmission^**^	405 (99.5)	379 (97.68)	**0.027**
Air-borne transmission	252 (61.92)	226 (58.25)	0.163
Contagion^**^	354 (86.98)	304 (78.35)	**0.001**
Fecal-oral transmission^***^	195 (47.91)	246 (63.40)	** <0.001**
Mother-baby transmission^**^	45 (11.06)	74 (19.07)	**0.001**
**Which masks do you think can obstruct SARS-CoV-2?^§^**	**4.92** ***±*** **0.60**	**4.74** ***±*** **1.10**	** <0.001** ^***^
N95	407 (100.0)	386 (99.48)	0.238
PM2.5 respirator	32 (7.86)	36 (9.28)	0.279
Sponge mask	14 (3.43)	10 (2.59)	0.308
Active carbon mask	5 (1.22)	8 (2.06)	0.259
Surgical mask	401 (98.52)	379 (97.68)	0.270
**Which ways do you think can inactivate SARS-CoV-2 effectively?^§^**	**3.50** ***±*** **2.29**	**2.47** ***±*** **2.50**	** <0.001** ^***^
Heating at 56°C for 30 min^***^	384 (94.35)	309 (79.64)	** <0.001**
75% ethyl alcohol^***^	399 (98.03)	355 (91.49)	** <0.001**
Chlorine-containing disinfectant^***^	322 (79.11)	242 (62.37)	** <0.001**
Chlorhexidine^**^	89 (21.86)	117 (30.15)	**0.005**
Ultraviolet radiation^***^	295 (72.48)	206 (53.09)	** <0.001**
**What are the initial manifestations of COVID-19?^§^**	**3.09** ***±*** **2.43**	**1.37** ***±*** **2.23**	** <0.001** ^***^
Fever, weakness and dry cough^***^	406 (99.75)	360 (92.78)	** <0.001**
Digestive symptoms, like nausea, vomiting, and diarrhea^***^	385 (94.59)	326 (84.02)	** <0.001**
Neurological symptoms, such as headache^***^	292 (71.74)	129 (33.24)	** <0.001**
Cardiovascular system symptoms, such as palpitation and chest tightness^***^	257 (63.14)	152 (39.18)	** <0.001**
Ophthalmic symptoms, such as conjunctivitis^***^	325 (79.85)	160 (41.24)	** <0.001**
Only mild limb or back muscle pain^***^	321 (78.87)	143 (36.86)	** <0.001**
**Which of the following specimens can detect nucleic acids of SARS-CoV-2?^§^**	**2.71** ***±*** **2.49**	**2.51** ***±*** **2.50**	0.092
Nasopharyngeal swab^***^	399 (98.03)	339 (87.37)	** <0.001**
Sputum^***^	384 (94.35)	306 (78.86)	** <0.001**
Secretion of lower respiratory tract^***^	363 (89.19)	299 (77.06)	** <0.001**
Blood	249 (61.18)	232 (59.79)	0.372
Feces^***^	357 (87.71)	288 (72.16)	** <0.001**
**What are the criteria for the release of isolation and discharge of patients?^§^**	**4.07** ***±*** **1.94**	**2.82** ***±*** **2.48**	** <0.001** ^***^
Temperature returns to normal for more than 3 days^***^	384 (94.34)	306 (78.86)	** <0.001**
Respiratory symptoms improved significantly^***^	341 (83.78)	237 (61.08)	** <0.001**
Pulmonary imaging shows obvious absorption of inflammation^***^	358 (87.96)	282 (72.68)	** <0.001**
The detection of respiratory pathogenic nucleic acid shows negative consecutive times (Sampling interval shall be at least 1 day)^***^	404 (99.26)	366 (94.32)	** <0.001**

§ *Multiple responses possible*.

* *p <0.05*.

** *p <0.01*.

*** *p <0.001*.

*Statistical significance (p <0.05) is indicated in bold. The questions and scores are also indicated in bold to distinguish it from proportion*.

**Table 3:** Results of multivariate analysis in mental status of medical staff.

**Variable**	**B**	**SE**	**β**	***t***	***p***	***R***	***R* ^**2**^**	**Adjusted *R*^**2**^**	**Durbin-Watson**
**Knowledge scores**
Constant	29.122	2.245		12.974	0.000^*^	0.426	0.181	0.174	1.939
Hospital level	−5.354	0.521	−0.400	−10.286	0.000^*^				
Education level	0.757	0.433	0.057	1.749	0.05^*^				
**SAR scores**
Constant	78.298	2.713		28.858	0.000^*^	0.477	0.228	0.224	1.914
Residence	2.711	0.775	0.113	3.498	0.000^*^				
Specialty	−6.445	1.028	−0.200	−6.269	0.000^*^				
Title	3.179	0.687	0.170	4.629	0.000^*^				
Education level	−7.891	0.678	−0.428	−11.633	0.000^*^				
**CES-D scores**
Constant	21.567	1.805		11.950	0.000^*^	0.361	0.130	0.126	1.841
Residence	1.601	0.516	0.106	3.106	0.002^*^				
Specialty	−4.971	0.684	−0.246	−7.269	0.000^*^				
Title	1.408	0.457	0.120	3.082	0.002^*^				
Education level	−2.434	0.451	−0.211	−5.394	0.000^*^				
**PSQI scores**
Constant	7.787	0.842		9.254	0.000^*^	0.242	0.058	0.055	1.694
Hospital level	1.051	0.239	0.152	4.408	0.000^*^				
Residence	0.689	0.251	0.096	2.748	0.006^*^				
Specialty	−1.328	0.335	−0.139	−3.961	0.000^*^				

* *p <0.05*.

#### Knowledge of Epidemiology

51.84 and 34.54% of the participants in group A and B individually knew SARS-CoV-2 is the correct name of the virus first-occurred in Wuhan. The pneumonia caused by SARS-CoV-2 was named as “COVID-19,” so nearly 57.48% confused these two concepts in group B (group A, 45.21%; *p* = 0.001). Droplet transmission, air-borne transmission and contagion were established ways of transmission. The scores of the distribution in group A was significantly higher than that in group B (2.79 ± 2.48 and 2.50 ± 2.50, *p* = 0.022), such as droplet transmission (99.5 and 97.68%, *p* = 0.026), contagion (86.98 and 78.35%, *p* = 0.001), fecal-oral transmission (47.91 and 63.40%, *p* < 0.001) and mother-baby transmission (11.06 and 19.07%, *p* < 0.001). Group A has a better understanding of effective SARS-CoV-2 inactivation methods than group B (3.50 ± 2.29 and 2.47 ± 2.50, *p* < 0.001), including heating at 56°C for 30 min (94.35 and 79.64%, *p* < 0.001), 75% ethyl alcohol (98.03 and 91.49%, *p* < 0.001), chlorine-containing disinfectant (79.11 and 62.37%, *p* < 0.001), and ultraviolet radiation (72.48 and 53.09%, *p* < 0.001).

#### Knowledge of Clinical Manifestations

Previous studies indicated the diversity of initial manifestations of COVID-19, including fever, weakness and dry cough, digestive symptoms (nausea, vomiting, and diarrhea), neurological symptoms (headache), cardiovascular system symptoms (palpitation and chest tightness), ophthalmic symptoms (conjunctivitis) as well as only mild limb or back muscle pain. Medical staff in group A had a better understanding of the above symptoms than group B (3.09 ± 2.43 and 1.37 ± 2.23, *p* < 0.001). When it came to the specimens that could detect nucleic acids of SARS-CoV-2, higher correct rates were found in group A, such as nasopharyngeal swab (98.03 and 87.37%, *p* < 0.001), sputum (94.35 and 78.86%, *p* < 0.001), secretion of lower respiratory tract (89.19 and 77.06%, *p* < 0.001), and feces (87.71 and 72.16%, *p* < 0.001). In addition, medical staff in tertiary hospitals had a more accurate grasp of the criteria for the release of isolation and discharge of patients than those in basic-level hospitals (4.07 ± 1.94 and 2.82 ± 2.48, *p* < 0.001).

### Degrees of Anxiety Among Chinese Medical Staff

**Figure 1:**
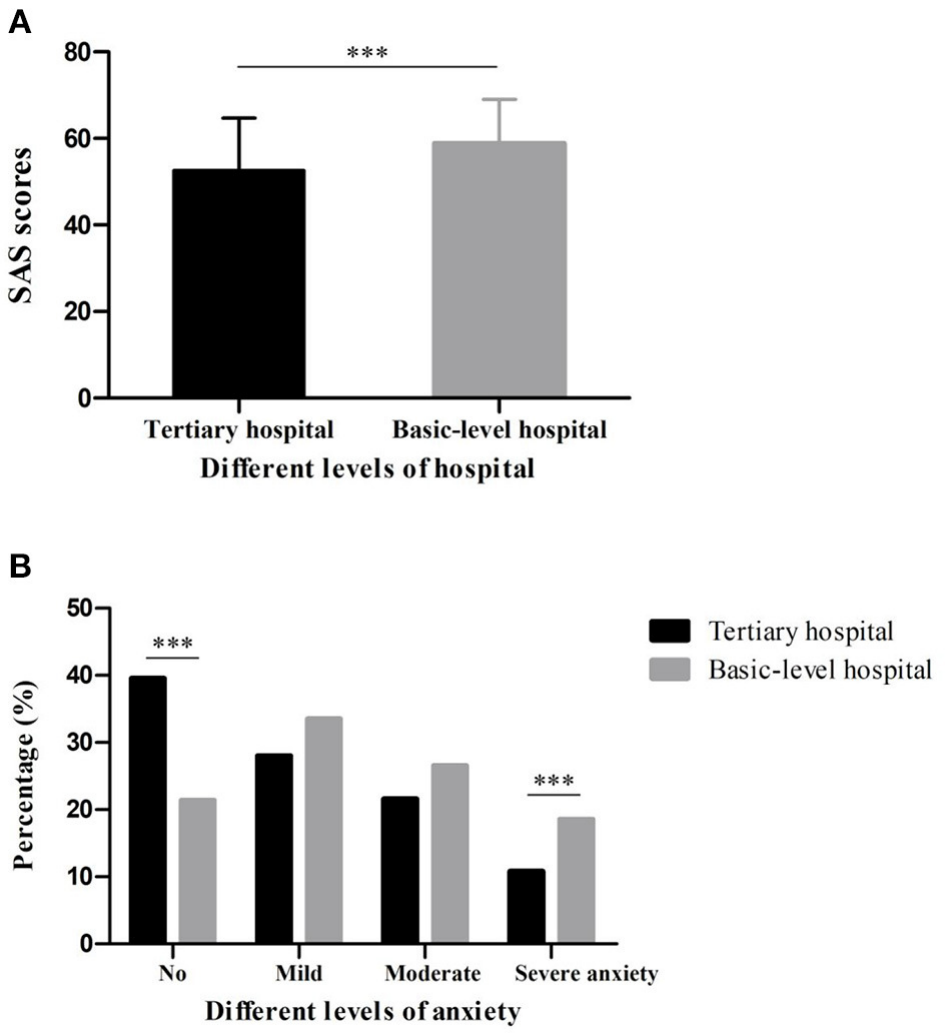
SAS scores **(A)** and different levels of anxiety **(B)** of medical staff in tertiary hospitals and basic-level hospitals during the break of COVID-19 (***p < 0.001).

Mild anxiety symptoms were found in both two groups. Furthermore, the SAS scores (Mean ± SD) among medical staff in basic-level hospitals were 58.87 ± 10.17, which was significantly higher than that of the tertiary hospitals group (52.59 ± 12.09, *p* < 0.001) (Figure 1A). Figure 1B showed that 39.56% of participants in group A and 21.39% in group B had no anxiety symptoms (*p* < 0.001). In addition, 18.56% of participants in group B had severe anxiety symptoms, with 10.81% of individuals in group A (*p* < 0.001). The multiple linear regression suggested four predictors could explain 22.4% of the SAS scores (*R*^2^ = 0.228, *F* = 58.12), including residence (β = 0.113, *p* < 0.001), specialty (β = −0.200, *p* < 0.001), title (β = 0.170, *p* < 0.001) and education level (β = 0.057, *p* = 0.05) (Table 3).

### Degrees of Depression Among Chinese Medical Staff

**Figure 2:**
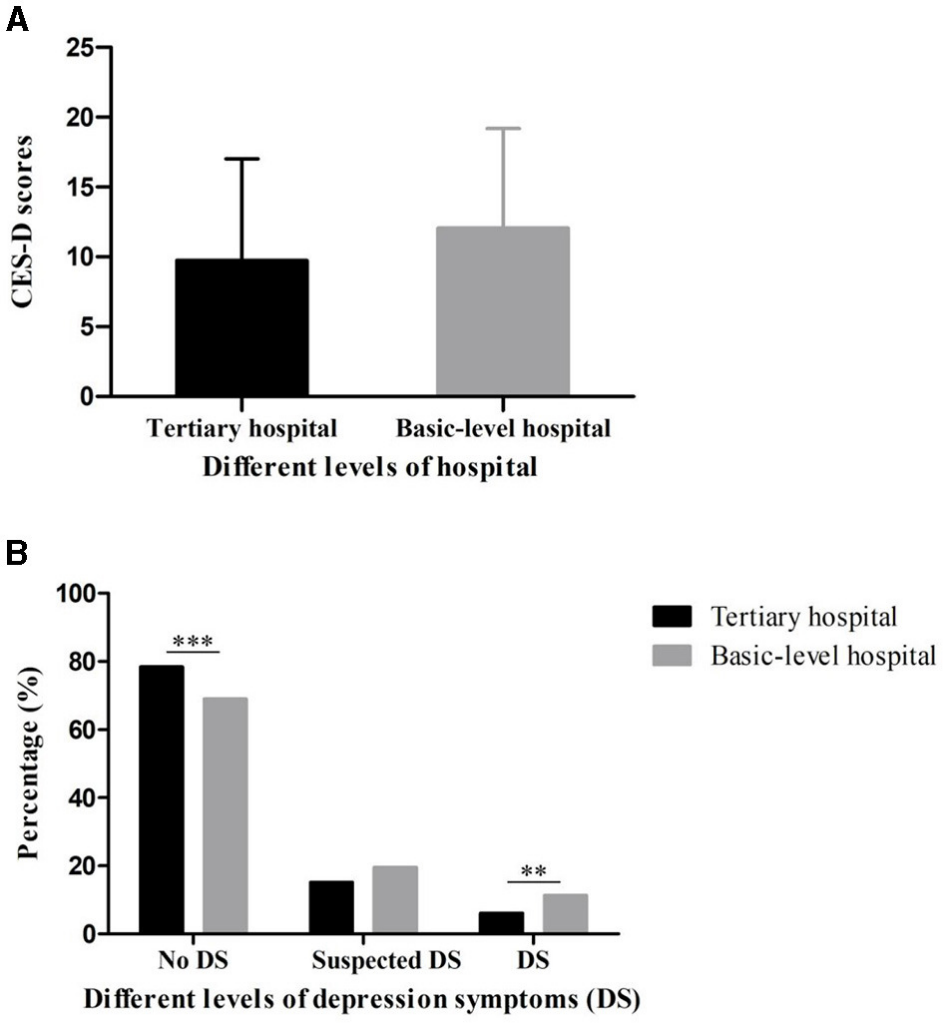
CES-D scores **(A)** and different levels of depression symptoms **(B)** of medical staff in tertiary hospitals and basic-level hospitals during the break of COVID-19 (***p < 0.001, **p <0.01).

As shown in Figure 2A, there were no significant differences in CES-D scores in group A (9.75 ± 7.26) and group B (12.05 ± 7.13) (*p* = 0.981). However, 78.43% individuals in group A had no depression symptoms, which was significantly higher than those in group B (69.07%, *p* < 0.001). Furthermore, definite depression symptoms were found in 6.13% participants in group A and 11.34% in group B (*p* = 0.006) (Figure 2B). The multiple linear regression suggested four predictors could explain 12.6% of the CES-D scores (*R*^2^ = 0.130, F = 29.48), including residence (β = 0.106, *p* = 0.002), specialty (β = −0.246, *p* < 0.001), title (β = 0.120, *p* = 0.002) and education level (β = −0.211, *p* < 0.001) (Table 3).

### Sleep Quality of Two Groups

**Figure 3:**
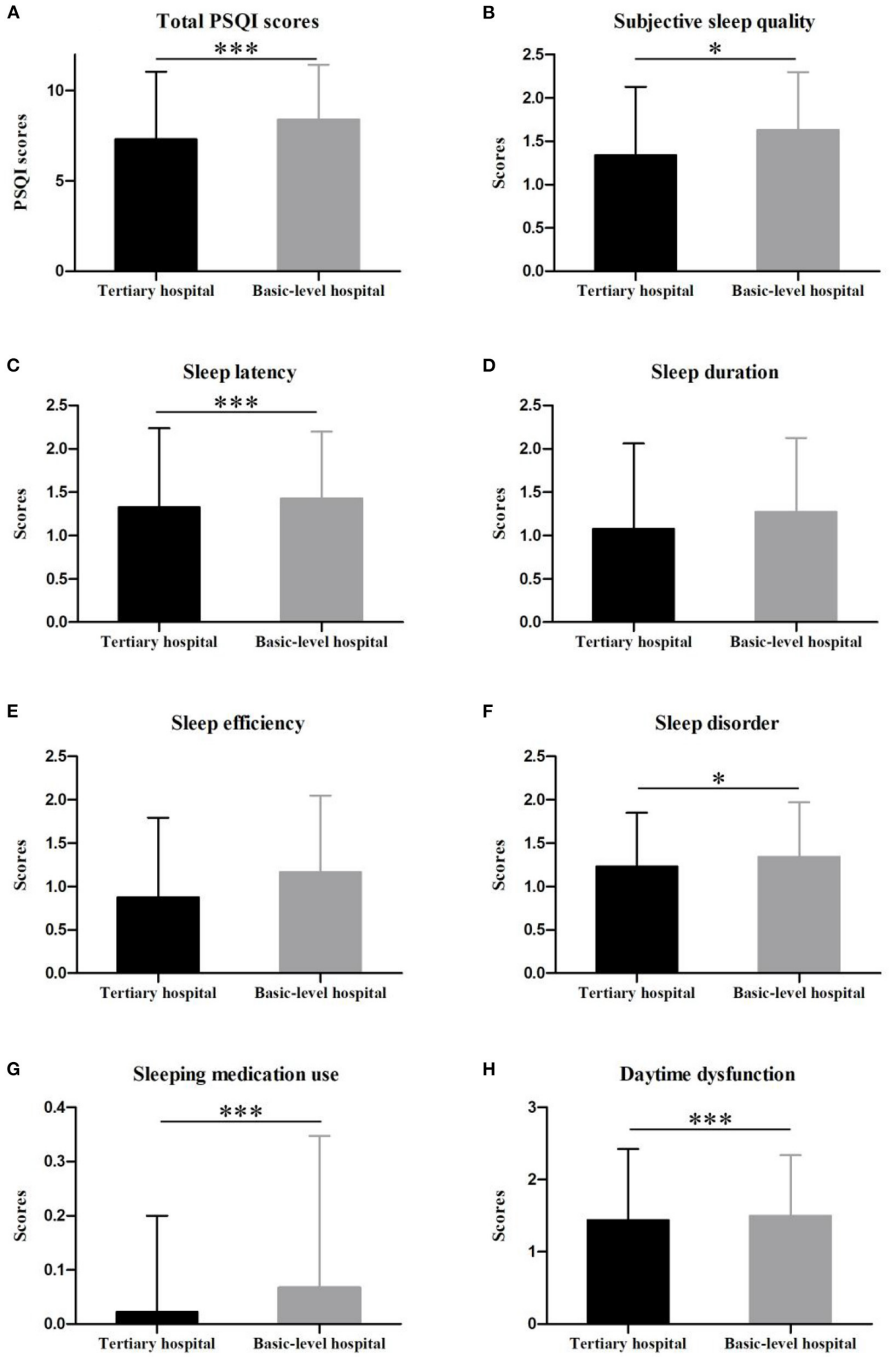
Comparison of groups in terms of the total Pittsburg Sleep Quality Scale Score **(A)** and subscale scores **(B–H)** (***p < 0.001, *p < 0.05).

The mean score of total PSQI among medical staff in basic-level hospital (8.41 ± 3.03) was statistically higher than that of participants in tertiary hospital (7.31 ± 3.74, *p* < 0.001) (Figure 3A). The scores of sub-components of group B, including subjective sleep quality, sleep latency, sleep disorder, sleeping medication use and daytime dysfunction, were significantly higher compared to Group A (*p* < 0.05) (Figures 3B–H). The multiple linear regression suggested three predictors could explain 5.5% of the PSQI scores (*R*^2^ = 0.058, *F* = 16.35), including hospital level (β = 0.152, *p* < 0.001), residence (β = 0.096, *p* = 0.006) and specialty (β = −0.139, *p* < 0.001) (Table 3).

## Discussion

This study was one of the first hospital-based attempts to investigate the knowledge, anxiety, depression and sleep quality of medical staff in China during the outbreak of COVID-19 and analyze whether they were associated with some demographic characteristics, especially the hospital levels. Our study showed greater anxiety, more severe depression and poorer sleep quality among medical staff in the central south areas of China. Additionally, compared to the tertiary hospital group, medical staff from basic-level hospitals had poorer knowledge and worse mental health conditions.

Previous studies demonstrated the prevalence of stress, anxiety, depression, and insomnia within not only the front-line healthcare workers caring for COVID-19 patients (30), but also medical staff working in their respective hospitals during the epidemic outbreak (31), which was consistent with our results. These psychiatry problems were closely related to numerous factors, such as the fear of contracting the disease and infecting family members, stressful shifts and little rest, leading to a state of psychological and physical tension capable of activating pathological behaviors (32, 33). These mental health problems affected the efficiency of fighting against COVID-19, as well as their overall well-being (34). Understanding the mental health response after public health emergencies could help medical staff and communities prepare for a population's response to an epidemic or a disaster (35). Therefore, it is important to control and prevent mental disorders of these medical staff for control of the epidemic and their long-term health. Some policies, measures and interventions have been taken in China to reduce the pressure on medical workers and address these mental disorders, such as establishing a shift system and online platforms with medical advice and identifying medical staff infected with COVID-19 while at work as work-related injuries (12, 36). It is worth mentioning that the National Health Commission of China published a national guideline of psychological crisis intervention for COVID-19 on 27th January, 2020, which was the first to initiate the guidance to provide multifaceted psychological protection of the mental health of medical staff in China (36).

Some demographic characteristics have been found to be related with health workers' knowledge and attitudes toward a certain disease, including age, title, education level, and hospital levels (14–16). Yang et al. indicated the knowledge and response to seizures among medical staff in tertiary hospitals were better than those of basic-level hospitals (18). Interestingly, we found the level of hospitals may also affect their health workers' understanding of COVID-19 during the epidemic outbreak. In China, medical resources are unevenly distributed, such as resources of equipment and talents, and are mainly concentrated in developed cities and high-level hospitals (37). We considered the medical curiosity as the main cause of the significant difference in the knowledge toward COVID-19 between the participants from tertiary and basic-level hospitals, which has been reported in recent studies (38, 39). Interestingly, studies of educational psychology revealed that the trait of curiosity is positively associated with academic achievement and the educational process may affect the state of curiosity of medical students (40). So how to maintain the medical curiosity is one of the main problems faced by the modern medical education. Only by being curious about their own abilities, can medical staff maintain a state of rapidly evolving medical knowledge and skills (41). Furthermore, medical education in China has emphasized the importance of treatment more than prevention for a long time. Specifically, the proportion of public health courses is relatively small in the current system of clinical medical education, and there are few opportunities for clinical medical students to participate in public health practice (22, 42). As a result, many medical workers were infected unexpectedly in the early stage of the epidemic, due to the insufficient public health literacy, especially in the basic-level hospitals (43). So it is important to expand the training of epidemic prevention talents and strengthen the teaching management of public health and preventive medicine, not only in medical school, but also in basic-level hospitals (44).

Several studies showed the high level of occupational stress and burnout among nurses could lead to anxiety, depression, and insomnia (45). Moreover, the risks of the these psychiatric problems in healthcare-seeking nurses were influenced by age, gender, job tenure, and hospital level (45). Nurses working in regional and local hospitals had higher hazard ratios for these psychiatric problems than the medical center group. Similarly, compared to the tertiary hospital group, greater anxiety, more severe depression and poorer sleep quality were found in medical staff from basic-level hospitals during the early stage of COVID-19. The different workloads and stressors among different hospital levels maybe the main reason for this finding. In addition, the discrepancies in the accessibility of help and barriers to help-seeking among different hospital levels maybe another possible explanation (46). In fact, the fear of the unknown could lead to high anxiety levels in both healthy people and people with preexisting mental health problems (47). The poorer knowledge of COVID-19 may explain the worse mental status among medical staff from basic-level hospitals. They did not know how to deal with patients unwilling to be quarantined at the hospital or did not cooperate with medical measures. However, Milgrom et al. found the anxiety scores among internal medicine residents were not a function of hospital level (48). The different epidemic situation in different regions may explain this discrepancy. Importantly, more attention should be paid to early identification and clinical psychological interventions of symptoms of anxiety and depression in susceptible medical staff from the basic-level hospitals during the epidemic (6, 49).

However, there are some limitations in our study that must be acknowledged. The focus on central south areas of China cannot represent the mental health of the entire population of medical staff from tertiary hospitals and basic-level hospitals individually. Next, there was not a cut-off value in the knowledge self-reported questionnaire, meaning that we could only compare the two sets of data to analyze whether there was a significant difference. Furthermore, the sample size is relatively small, which we hope to expand in future work. Also, the participants may be worried about the confidentiality of this study since it was conducted by their peers, which may have an impact on their responses.

## Conclusion

This study was one of the first hospital-based attempts to investigate the knowledge, anxiety, depression, and sleep quality of medical staff in China. Greater anxiety, more severe depression and poorer sleep quality were found among medical staff in central south areas of China during the COVID-19 outbreak. Additionally, compared to the tertiary hospital group, medical staff from basic-level hospitals had poorer knowledge of COVID-19 and worse mental health conditions, which might further affect the efficiency of fighting against COVID-19 and their overall well-being. In addition, residence, specialty, title, and education level may also be factors of knowledge of COVID-19 and psychiatry problems. In light of this information, more attention should be paid to early identification and intervention of symptoms of anxiety and depression in susceptible medical staff from the basic-level hospitals.

## Data Availability Statement

The raw data supporting the conclusions of this article will be made available by the authors with requirement.

## Ethics Statement

The studies involving human participants were reviewed and approved by the Ethics Committees of the Xiangya Hospital, Central South University. The patients/participants provided their written informed consent to participate in this study.

## Author Contributions

The idea was conceived by HY, RS, and HW. HY, RS, and LF were responsible for the writing and revising manuscript. All authors were involved in the data collecting and statistics. All authors contributed to the article and approved the submitted version.

## Funding

This study was supported by the National Natural Science Foundation of China (81771407 & 82071461).

## Conflict of Interest

The authors declare that the research was conducted in the absence of any commercial or financial relationships that could be construed as a potential conflict of interest.

## Publisher's Note

All claims expressed in this article are solely those of the authors and do not necessarily represent those of their affiliated organizations, or those of the publisher, the editors and the reviewers. Any product that may be evaluated in this article, or claim that may be made by its manufacturer, is not guaranteed or endorsed by the publisher.
